# Label-free nonlinear optical microscopy detects early markers for osteogenic differentiation of human stem cells

**DOI:** 10.1038/srep26716

**Published:** 2016-05-26

**Authors:** Arne D. Hofemeier, Henning Hachmeister, Christian Pilger, Matthias Schürmann, Johannes F. W. Greiner, Lena Nolte, Holger Sudhoff, Christian Kaltschmidt, Thomas Huser, Barbara Kaltschmidt

**Affiliations:** 1 Cell Biology, University of Bielefeld, D-33501 Bielefeld, Germany; 2 Biomolecular Photonics, University of Bielefeld, D-33501 Bielefeld, Germany; 3 Department of Otolaryngology, Head and Neck Surgery, Klinikum Bielefeld, D-33604 Bielefeld, Germany; 4 Molecular Neurobiology, University of Bielefeld, D-33501 Bielefeld, Germany

## Abstract

Tissue engineering by stem cell differentiation is a novel treatment option for bone regeneration. Most approaches for the detection of osteogenic differentiation are invasive or destructive and not compatible with live cell analysis. Here, non-destructive and label-free approaches of Raman spectroscopy, coherent anti-Stokes Raman scattering (CARS) and second harmonic generation (SHG) microscopy were used to detect and image osteogenic differentiation of human neural crest-derived inferior turbinate stem cells (ITSCs). Combined CARS and SHG microscopy was able to detect markers of osteogenesis within 14 days after osteogenic induction. This process increased during continued differentiation. Furthermore, Raman spectroscopy showed significant increases of the PO_4_^3−^ symmetric stretch vibrations at 959 cm^−1^ assigned to calcium hydroxyapatite between days 14 and 21. Additionally, CARS microscopy was able to image calcium hydroxyapatite deposits within 14 days following osteogenic induction, which was confirmed by Alizarin Red-Staining and RT- PCR. Taken together, the multimodal label-free analysis methods Raman spectroscopy, CARS and SHG microscopy can monitor osteogenic differentiation of adult human stem cells into osteoblasts with high sensitivity and spatial resolution in three dimensions. Our findings suggest a great potential of these optical detection methods for clinical applications including *in vivo* observation of bone tissue–implant-interfaces or disease diagnosis.

Minimally invasive assessment of the degree of differentiation of cells derived from embryonic or adult stem cells is a topic of particular interest in regenerative medicine. Most current biochemical analysis methods consist of a combination of immunostaining, reverse transcription polymerase chain reaction (rtPCR), protein blotting assays, mass spectrometry and a few other means, which are either destructive to or contaminate stem cells, rendering them impossible for use in therapeutic applications. Consequently, the development of minimally invasive and nondestructive analysis methods that can provide similar sensitivity as traditional analysis methods while maintaining their therapeutic potential are in high demand. Raman spectroscopy is a label-free optical analysis method that can assess the biochemical content of cells without destroying or contaminating the cells of interest, which could readily meet this need^1^,^2^,^3^,^4^. Raman scattering is the inelastic scattering of photons by molecular bonds. In a sample of interest, photons from a monochromatic source, typically a narrow-band laser, can interact with molecular bonds in two ways. In most cases they induce the excitation of a molecular bond vibration and the resulting loss of this energy is measured in the scattered photon (Stokes scattering). The other, more rare event, is that an existing bond vibration can transfer its energy to the scattered photon resulting in the detection of a blue-shift of the photon wavelength (anti-Stokes scattering). Analyzing the change in energy, or the frequency shift of the scattered light provides information about the nature of the chemical bond and its chemical microenvironment. The nature of the Raman scattering process makes spontaneous Raman scattering rather inefficient and requires extended signal integration times which are unsuitable for many live-cell imaging applications.

One derivative designed to overcome the low signal issue of conventional Raman spectroscopy is coherent anti-Stokes Raman scattering (CARS)^5^. When implemented as an imaging technique, CARS microscopy enables live-cell imaging by utilizing a four-wave mixing process in order to detect a specific molecular bond vibration in a pump-probe experiment^4^,^6^. Here, two incoming pump photons with frequency *ω*_*p*_ and the probe photon with frequency *ω*_*S*_ coherently excite the resonant molecular bond vibrations collectively, leading to a strong anti-Stokes signal at frequency *ω*_*AS*_ = 2*ω*_*p*_ − *ω*_*S*_. The resulting anti-Stokes signal is up to 5 orders of magnitude stronger than the corresponding spontaneous Raman scattering signal^4^. Due to the increased signal strength CARS data can be collected pixel-by-pixel, while scanning across a biological sample to enable chemically specific imaging, even at video-rate^6^. Exciting the CARS process in the near-infrared wavelength range enables one to combine this process with other nonlinear optical imaging mechanisms, such as two-photon excited fluorescence, as well as second harmonic generation (SHG).

SHG is the conversion of two photons of the same wavelength into a single photon at half the wavelength of the original photons. This second-order process requires a break of centrosymmetric symmetry, which typically occurs at interfaces, in specific birefringent crystals, or at highly ordered crystalline regions within biological samples, such as collagen type 1 fibers^7^. The resulting blue-shifted signal is, again, spectrally extremely narrow and can readily be detected by the use of suitable filter sets.

Raman spectroscopy, both in its spontaneous and coherent implementations has been increasingly used to characterize stem cells and their derivatives during the last decade^8^. Notingher and coworkers were among the first to analyze murine embryonic stem cells and established markers to assess their differentiation by multivariate analysis of single cell Raman spectra^9^. The same group demonstrated the specific detection of Raman signatures of different bone cell phenotypes^10^. Adult mesenchymal stem cells from rhesus monkey and their derivative were further successfully analyzed by Raman spectroscopy^11^. Time-lapse Raman spectroscopy was successfully applied to image the differentiation of murine osteoblasts^12^. Extending these promising findings to the human system, Chan *et al.* reported the successful application of Raman spectroscopy to separate human embryonic stem cells and their cardiac derivatives^13^. Coherent Raman scattering was first used in 2007 to analyze and image living embryonic stem cells^14^. Later, blastocysts^15^, and most recently, in the form of broadband CARS microscopy, coherent Raman scattering was applied to characterize and image human mesenchymal stem cells and their differentiation into adipocytes and osteoblasts^16^. Particularly the strong vibrational modes of mineral deposits have been shown to enable the successful monitoring of osteogenic differentiation of human mesenchymal stem cells into osteoblasts based on spontaneous Raman spectroscopy^17^,^18^,^19^.

In the present study, we used a combination of spontaneous Raman spectroscopy as well as CARS and SHG microscopy to assess and visualize the osteogenic differentiation of adult human neural crest-derived stem cells from the inferior turbinate of the human nasal cavity. Inferior turbinate stem cells (ITSCs) are routinely isolated during minimally-invasive surgery^20^ and can easily be expanded *in vitro*^21^ under clinical-grade conditions^22^. *In vitro*, ITSCs revealed an extraordinarily broad differentiation potential into ectodermal as well as mesodermal cell types^21^,^23^,^24^. By applying a chemically-defined directed differentiation medium or topological cues of a nanoporous titanium surface, we particularly showed the differentiation of ITSCs into osteogenic derivates^21^,^24^. Here, CARS microscopy was used to visualize the cells by their lipid signal, to determine the biomineralization of calcium hydroxyapatite in differentiated cells, and SHG enabled us to detect collagen secreted by osteogenically differentiated ITSCs. The fact that we could detect some of these marker signals simultaneously with traditional biochemical analyses provided discernible results. Our findings thus indicate that these label-free analysis methods are applicable to monitoring osteogenic differentiation of adult human stem cells, suggesting their potential use for future clinical applications.

## Results

**Figure 1:**
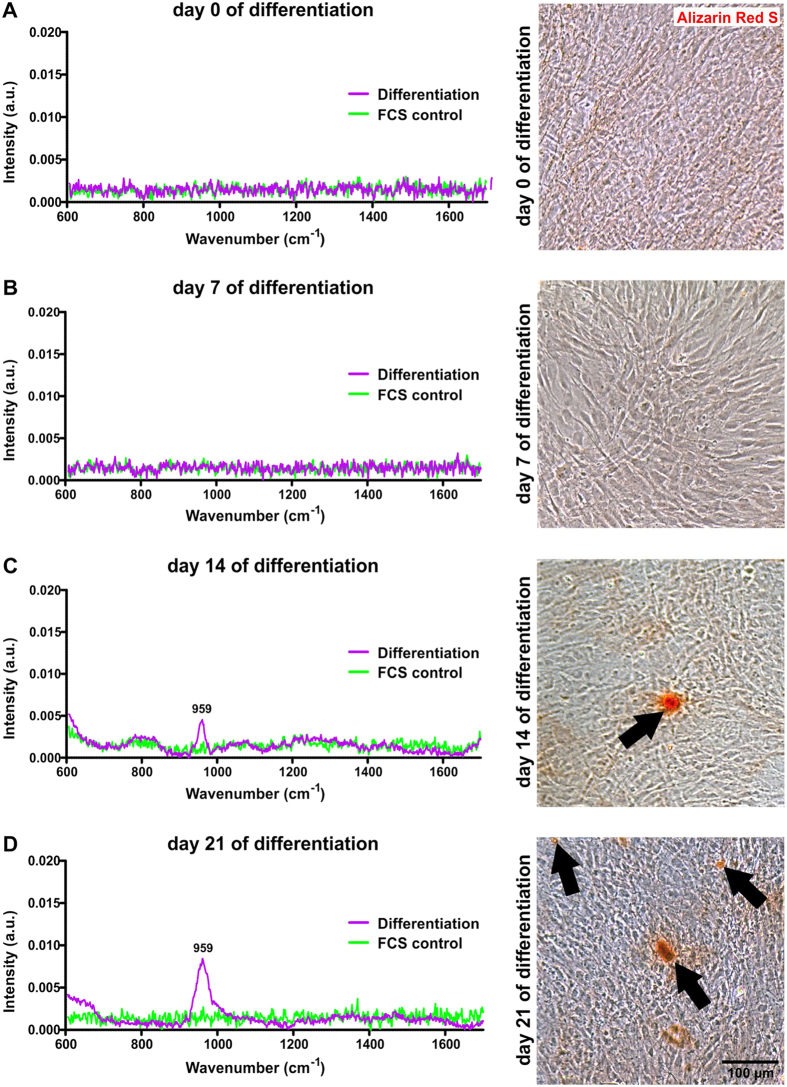
Raman spectroscopy successfully shows calcium deposition occurring during osteogenic *in vitro* differentiation of nasal stem cells. (**A,B**) During early osteogenic differentiation, inferior turbinate stem cells showed no signs of characteristic Raman spectra or Alizarin Red S-stained calcium deposition (arrows). Cells treated with medium containing fetal calf serum (FCS) without differentiation supplements served as negative control (FCS control) for the differentiation approach. (**C,D**) After 14 and 21 days of directed differentiation, ITSCs revealed calcium deposits exhibiting distinct peaks of Raman spectra (averages of three spectra each) at 959 cm^−1^, which were also Alizarin Red S-positive.

**Figure 2:**
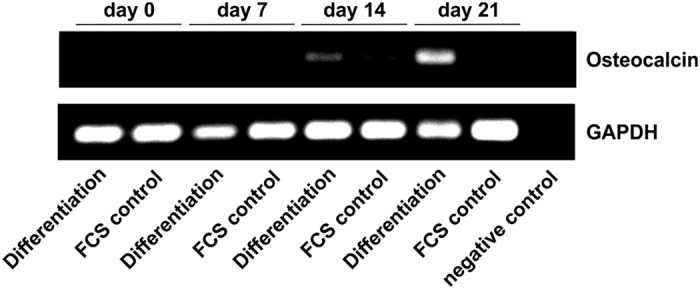
Characteristic expression of Osteocalcin validates successful osteogenic differentiation of ITSCs. RT-PCR showed characteristic expression of Osteocalcin in ITSCs after 14 and 21 days of directed osteogenic differentiation (differentiation) in contrast to ITSCs treated with medium containing fetal calf serum in the absence of differentiation supplements (FCS control). Non template control served as negative control and Glyceraldehyde 3-phosphate-Dehydrogenase (GAPDH) as house keeping gene.

In order to induce osteogenic differentiation in ITSCs, we applied a biochemically defined differentiation medium containing dexamethasone, β-glycerophosphate and phosphate in accordance to our previous studies^20^,^21^. During the first seven days of differentiation, Raman spectroscopy followed by Alizarin Red staining of the same quartz cover slip revealed no signs of calcium hydroxyapatite deposition in ITSCs (Fig. 1A,B). Notably, Raman spectroscopy displayed a characteristic 959 cm^−1^ peak assigned to the PO_4_^3−^ symmetric stretching vibration of calcium hydroxyapatite in the osteogenically induced cells after 14 and 21 days of directed differentiation (Fig. 1C,D), whereas no distinct spectra were observable in control cells (Fig. 1C,D). Accordingly, Alizarin Red staining subsequently applied after Raman spectroscopy revealed characteristic calcium deposition in ITSCs differentiated for 14 and 21 days (Fig. 1C,D, arrows) in contrast to the control approach (Fig. S1). Reverse transcription PCR analysis further confirmed the successful osteogenic differentiation of ITSCs at the mRNA level by revealing expression of the characteristic osteogenic marker osteocalcin after 14 and 21 days of differentiation (Fig. 2).

**Figure 3:**
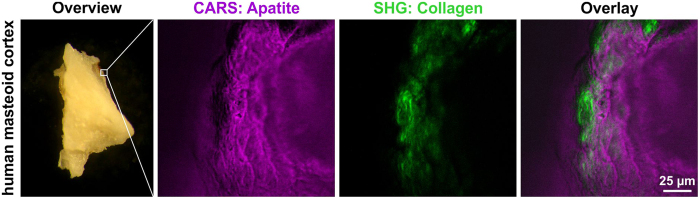
Successful imaging of hydroxyapatite and collagen in human skull bone using CARS and SHG microscopy. Human mastoid cortex showed a CARS signal (magenta) for calcium hydroxyapatite (959 cm^−1^) as well as an SHG signal (532 nm) for collagen (green).

**Figure 4:**
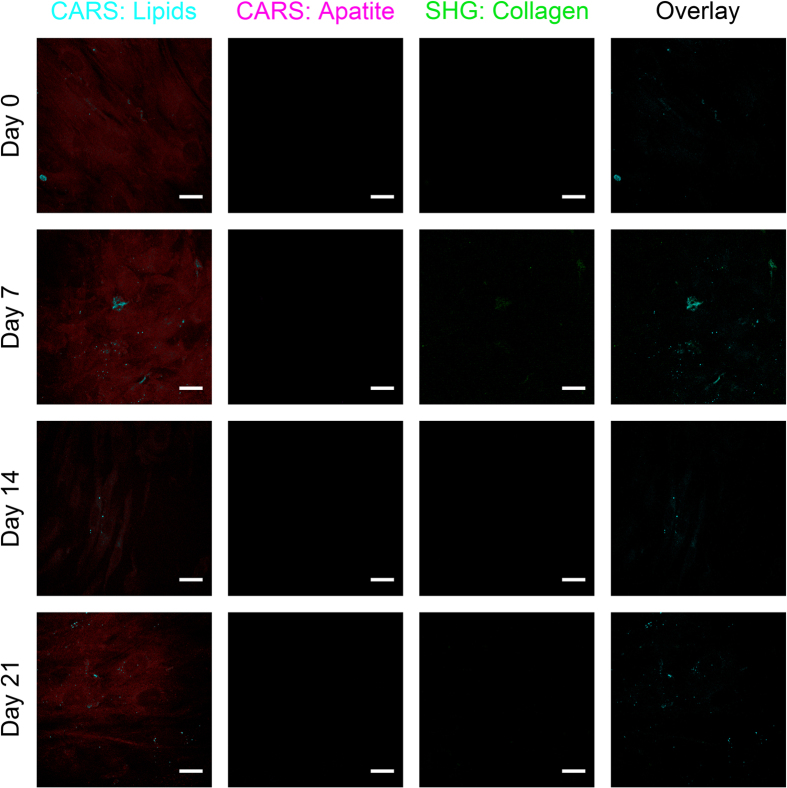
ITSCs cultivated under control conditions show no signs of calcium hydroxyapatite deposition or collagen detectable with CARS and SHG imaging. ITSCs cultivated for 21 days in medium containing FCS but no differentiation supplements showed no CARS signal (magenta) for calcium hydroxyapatite (959 cm^−1^) and no SHG signal (green, 532 nm). CARS images obtained at the lipid resonance (2845 cm^−1^) provide contrast for ITSCs by local lipid accumulations (cyan), as well as the non-resonant background (dark red). Scale bar: 20 μm.

**Figure 5:**
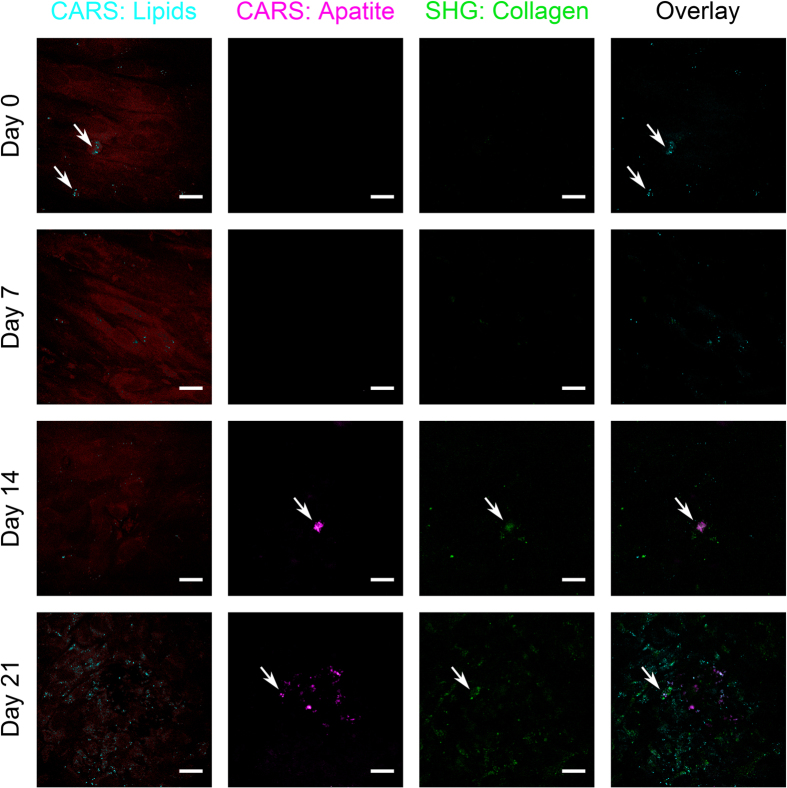
CARS and SHG imaging reveal calcium hydroxyapatite deposits and collagen after osteogenic differentiation of ITSCs. No calcium hydroxyapatite deposits or collagen was detectable by CARS (959 cm^−1^, magenta) and SHG (532 nm, green) microscopy at the start of directed osteogenic differentiation of ITSCs (d 0, d 7). After 14 and 21 days of directed differentiation of ITSCs, SHG microscopy and CARS revealed increasing amounts of calcium hydroxyapatite (magenta) and collagen (green) (d 14, d 21). Scale bar: 20 μm.

In addition to Raman spectroscopy, the non-invasive and label-free multimodal imaging approaches CARS and SHG microscopy were simultaneously applied to detect and characterize osteogenic differentiation. We initially validated our CARS and SHG microscopy setups by successfully imaging of collagen in the mouse tail (Fig. S2) as well as of calcium hydroxyapatite and collagen in native human and mouse skull bone (Fig. 3, Fig. S3). To visualize ITSCs, the confluent cell layer was imaged by CARS microscopy probing the 2845 cm^−1^ CH_2_ vibration of lipids, resulting in a specific contrast provided by lipids present within the cultivated cells (Fig. 4). Because of the rather weak and highly localized occurrence of lipid CARS signals, the simultaneously occurring non-resonant CARS signal is used to display the confluent cell layer (shown in deep red), while lipid accumulations are shown in a different color (cyan). CARS microscopy was also used to probe the PO_4_^3−^ stretching mode at 959 cm^−1^ to visualize calcium hydroxyapatite deposition (magenta), while SHG microscopy was used to analyze the collagen secretion (green) of ITSCs. Neither SHG, nor CARS revealed significant signals during cultivation of ITSCs for 21 days under control conditions in medium containing FCS without differentiation supplements (Fig. 4). Notably, CARS microscopy probing the PO_4_^3−^ stretching mode at 959 cm^−1^ revealed the presence of calcium hydroxyapatite deposits in differentiated ITSCs after 14 days. Co-localized to the CARS-signal, we were able to detect collagen type I by simultaneously applying SHG microscopy (Fig. 5, arrows). After 21 days of differentiation, several calcium hydroxyapatite deposits were observed in ITSCs using CARS microscopy. In addition, collagen type I was ubiquitously observable in the culture via SHG microscopy, which was co-localized to calcium hydroxyapatite deposits visualized by CARS (Fig. 5, arrows).

**Figure 6:**
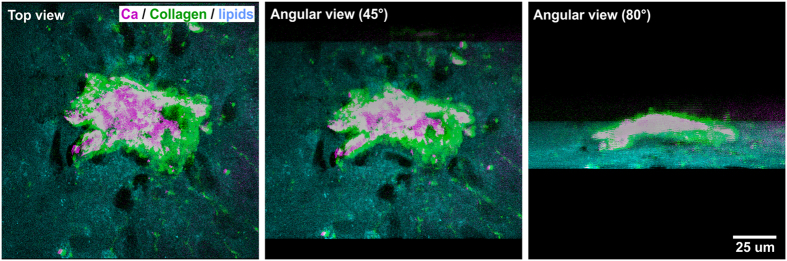
CARS and SHG microscopy reveal three-dimensionality of ITSC-derived calcium hydroxyapatite deposits embedded in collagen. After 21 days of directed osteogenic differentiation of ITSCs, three dimensional calcium hydroxyapatite (Ca) deposits were observable using CARS. SHG microscopy further revealed the surrounding collagen matrix.

To further determine the structure of the co-localized collagen and calcium hydroxyapatite deposits in more detail, we simultaneously acquired three-dimensional (3D) CARS and SHG images of a large hydroxyapatite crystal, by taking consecutive CARS and SHG images at 26 different z-sections followed by 3D-reconstruction in the open source image processing package Fiji. Notably, we observed the mineral deposit to be localized external and superior to the cell layer, accompanied by heavy collagen secretion resulting in a surrounding collagen matrix (Fig. 6).

## Discussion

In the current study we demonstrate the already described great osteogenic differentiation potential of ITSCs^20^,^21^ by Alizarin Red staining and PCR analysis. Although commonly applied to analyze osteogenic differentiation, these methods are invasive due to fixation or cell disruption. Facing this challenge, we were able to show that Raman spectroscopy alone is sufficient to monitor the development auf calcium apatite *in vitro* and over time. This finding is in accordance with other studies utilizing Raman spectroscopy to successfully detect osteogenic differentiation^8^,^11^,^12^,^13^,^20^. McManus *et al.* reported the detection of the 959 cm^−1^ PO_4_^3−^ symmetric stretching vibration of calcium hydroxyapatite by Raman spectroscopy during osteogenic differentiation of human mesenchymal stem cells^18^. In our study, the 959 cm^−1^ vibration initially appeared in Raman spectra on day 14 after osteogenic induction of ITSCs and a further increased intensity was detectable seven days later.

In addition to the detection of osteogenic differentiation by spontaneous Raman spectroscopy we successfully applied SHG and CARS to obtain microscopic images of the differentiation process. The high sensitivity and specificity of SHG microscopy to collagen type I was already investigated in tissue samples. Strubler and coworkers could easily discriminate between type IV and type I collagen^7^, because type IV generates a vanishingly low SHG signal due to its mesh like structure. Interestingly Cox and colleagues could show that SHG signal of fibrils formed from collagen type III is much weaker compared to that of collagen type I, which might be based on the less crystalline structure of type III collagen.

Our results demonstrate that SHG microscopy is also sufficient to detect collagen type I secretion from osteogenic cells in culture. This is of particular interest, since collagen secretion serves as hallmark for osteogenic differentiation. Furthermore, the distribution of the secreted collagen type I plays a key role in the further development of osteogenic phenotype *in vitro*^25^. CARS microscopy has been utilized to image different biological specimens (reviewed in^26^). Here, we show that CARS microscopy can serve as a useful tool to monitor calcium deposits containing high amounts of PO_4_^3−^ as a further marker of osteogenic differentiation of ITSCs. Other groups used broadband CARS to detect calcium apatite during osteogenic differentiation of mesenchymal stem cells^16^. In addition, the simultaneous detection of the SHG and CARS signal allows investigations regarding the co-localization of collagen and calcium apatite.

Furthermore, by applying 3D imaging and image reconstruction, we were able to study the three-dimensional structure and composition of calcium deposits. Using an entirely non-destructive imaging process we found that the mineralized portion of the deposits was surrounded by a dense collagen matrix. This structure and composition of calcium deposits present in osteogenic cultures is in good accordance with a study by Davies and coworkers. Utilizing electron microscopy, the authors investigated the presence and structure of initial calcified globular accretions made by differentiating osteogenic cells^27^. Synthesis of collagen by the differentiating osteogenic cells is followed by collagen fiber assembly and mineralization, which is in accordance to our observations.

In summary, this study emphasizes the significant potential of CARS and SHG microscopy in addition to Raman spectroscopy for the detection of osteogenic differentiation and promotes these label-free approaches for future *in vivo* investigations.

## Conclusions

In our present study, we established Raman spectroscopy as well as CARS and SHG microscopy for the detection of osteogenic differentiation of adult human neural crest-derived stem cells. Furthermore, we demonstrated the great capability of ITSCs to differentiate into osteoblasts *in vitro*, providing an enhanced potential for tissue regeneration during injuries or for autologous stem cell therapies treating osteodegenerative diseases in the future. Imaging the continued differentiation process by 3D CARS and SHG microscopy showed that hydroxyapatite crystals rapidly form outside the ITSC layer and are surrounded by a collagen network.

These findings imply that the non-destructive and label-free analysis approaches provided by Raman spectroscopy, in combination with multimodal imaging methods, such as CARS and SHG microscopy, provide similar sensitivities as destructive biochemical methods for the analysis of stem cell differentiation, with the added benefit of providing structural information by microscopy. These methods are readily extendable for clinical applications even *in vivo,* such as the endoscopic observation of bone tissue-implant-interfaces. In addition, the here presented techniques may facilitate the diagnosis of ossification-related diseases in terms of bone mineralization density distribution without taking respective biopsies from patients.

## Materials and Methods

### Human material

Human material was obtained by biopsy during routine surgery after informed consent according to local and international guidelines. The ethics board of the medical faculty of the University of Münster approved of all the procedures described in this article (No. 2012–015-f-S). All experiments were performed in accordance with these approved guidelines and regulations.

### Mouse material

Mouse tissue was extracted according to local guidelines from the LANUV, NRW. All the procedures concerning mouse material described in this article were approved by the LANUV, NRW. All experiments were performed in accordance with these approved guidelines and regulations. C57BL/6 mice were euthanized followed by cervical dislocation. Skull bones and tail of mice were removed and retained in PBS (1x).

### Isolation and culturing of inferior turbinate stem cells (ITSCs)

Human nasal inferior turbinates were obtained by biopsy during routine surgery after informed consent according to local and international guidelines. The ethics board of the medical faculty of the University of Münster approved of all the procedures described in this article (No. 2012–015-f-S). All experiments were performed in accordance with these approved guidelines and regulations. ITSCs were isolated from adult human inferior turbinate tissue and pre-cultured as previously described by Hauser *et al.*^20^. The isolated ITSCs were cultured within a 3D blood plasma matrix as described by Greiner *et al.*^21^. Blood plasma was kindly provided by the Institut für Laboratoriums- und Transfusionsmedizin (Herz- und Diabeteszentrum NRW, Bad Oeynhausen, Germany). Briefly, Dulbecco’s modified Eagle’s medium/Ham’s F-12 (1:1) (DMEM/F-12; Biochrom, Berlin, Germany) containing L-Glutamin (L-Glu; 200 mM; Sigma-Aldrich, St. Louis, USA), epidermal growth factor (EGF; 20 ng/mL; R&D Systems, Wiesbaden, Germany), basic fibroblast growth factor (bFGF/FGF-2; 40 ng/mL; lab-made) and B27 supplement^28^ was applied, hereinafter referred to as standard medium. The cells were cultured in tissue culture flasks (TPP, Trasadingen, Austria) in standard medium containing 10% blood plasma at 37 °C, 5% O_2_ and 5% CO_2_ in a humidified incubator (Binder, Tuttlingen, Germany) and fed every two to three days. For passaging, collagenase I (Serva Electrophoresis, Heidelberg, Germany) was applied for one hour at 37 °C followed by harvesting by centrifugation at 300 × g (Universal 320, Hettich GmbH, Tuttlingen, Germany) and cultivation in standard medium with 10% blood plasma as described above.

### Osteogenic differentiation of ITSCs

For forced osteogenic differentiation, ITSCs were seeded in DMEM containing 10% fetal calf serum (FCS, Sigma-Aldrich, St. Louis, USA) at *a density* of 1 × 10^5^/cm^2^ on different quartz cover slips and at 3 × 10^3^/cm^2^ in different six well plates (Sarstedt, Nümbrecht, Germany). At a confluency of approximately 80%, osteogenic induction medium was applied containing DMEM, 10% FCS, 100 nM dexamethasone (Sigma-Aldrich, St. Louis, USA), 10 mM *β* -glycerophosphate (Sigma-Aldrich, St. Louis, USA) and 0.05 mM L-ascorbic acid-2-phosphate (Sigma-Aldrich, St. Louis, USA) at 37 °C and 5% CO_2_ in a humidified incubator as described by Greiner *et al.*^21^. Control cells were cultured in DMEM supplemented with 10% FCS. Cells were fed every three to four days. Differentiated and control cells cultured on quartz cover slides were imaged by CARS microscopy, probed by local Raman spectra, and the same quartz cover slides were analyzed via Alizarin Red S staining. Cells cultivated in six well plates were analyzed via RT-PCR. Spontaneous Raman spectra are influenced by the choice of the substrate, because all materials within the extended laser focus will generate a Raman signal. Regular (BK7) glass slides are not suitable due to the strong autofluorescence of BK7 glass when excited at 785 nm and its intrinsic Raman signature. We chose quartz for its robustness over other materials, such as MgF_2_ or CaF_2_, which are very brittle and difficult to handle for subsequent analyses, such as CARS.

### Reverse transcription PCR

Total RNA isolation was conducted using the RNeasy Mini Kit (QIAGEN, Hilden, Germany) according to the manufacturer’s guidelines. Quality and concentration of the isolated RNA was determined by Nanodrop UV spectrophotometry. Thereafter, cDNA was synthesized using First Strand cDNA Synthesis Kit (Fermentas, St. Leon-Rot, Germany) followed by PCR using the GoTaq G2 DNA Polymerase Kit (Promega, Madison, USA) according to manufacturer’s guidelines. Primer sequences were CTGCACCACCAACTGCTTAG (forward primer GAPDH), GTCTTCTGGGTGGCAGTGAT (reverse primer GAPDH) as well as CCTTTGTGTCCAAGCAGGAG (forward primer Osteocalcin) and TCAGCCAACTCGTCACAGTC (reverse primer Osteocalcin).

### Raman spectroscopy

For spontaneous Raman spectroscopy, a custom-built Raman micro-spectroscopy setup was used. Quartz cover slips served as substrate for biological samples. Different cover slips were used for different time points of differentiation. A 785 nm continuous wave diode laser (Innovative Photonic Solutions, Monmouth Junction, New Jersey, USA) is the main light source for Raman spectroscopy. A Faraday rotator (Optics for research, Caldwell, New Jersey, USA) was placed behind the laser in order to prevent instabilities by directly back-reflected light followed by a 785 nm narrow-band clean-up filter (785/3, Semrock, Rochester, New York, USA). After the laser beam was reflected by a 785 nm dichroic long pass mirror (Razor Edge, Semrock, Rochester, New York, USA), a 60X Olympus UPLSAPO water immersion objective lens (1.2 NA; Olympus, Hamburg, Germany) was used to focus the beam into the sample. Under these conditions, a laser spot volume of <1 femtoliter with a laser power at the sample of <25 mW was achieved. The laser spot diameter was approximately 1 μm. Several sample locations were probed by spontaneous Raman spectroscopy with this spot diameter, guided by the transmitted light microscopy images of the samples, where the formation of hydroxyapatite crystallites was indicated based on the cell morphology. An indication for potential crystallite formation was provided by a star-shaped arrangement of cells according to^20^,^21^, the center of which was then probed by RS. Stokes-shifted Raman-scattered photons were collected by the same objective lens and passed the 785 nm dichroic mirror. A 785 nm dichroic long pass filter was used to remove residual laser light before the Raman signal was directed into a multimode step-index fiber (AFS 105/125Y, 0.22 NA, 105 μm, 400–2400 nm, Thorlabs, Dachau/Munich, Germany). The fiber was connected to a spectrograph (Acton 2300i, Princeton Instruments, Planegg/Martinsried, Germany) equipped with a 600 lines/mm grating, blazed at 1 μm wavelength, and a CCD camera (Newton, DU 920P BR-DD, Andor, Belfast, UK).

Prior to each Raman measurement the system was calibrated using an undiluted toluene solution as reference source. All Raman spectra were recorded in the range of 400 to 2000 cm^−1^ with an integration time of 30 seconds per spectrum.

Broadband background signals from sample and/or substrate, such as autofluorescence, was subtracted using a modified version of an automated background subtraction algorithm originally developed by Lieber *et al.*^29^.

### Histochemical staining (Alizarin Red Staining)

ITSCs differentiated for 0, 7, 14 and 21 days in osteogenic induction medium were fixed for 10 min utilizing 4% PFA For the Alizarin Red S staining, 1% Alizarin Red (Waldeck, Müster, Germany) in ddH_2_O with a pH value of 4.3 was applied for 15 min at RT. The cells were imaged with the AMG EVOS xl microscope (PeqLab, Erlangen, Germany).

### CARS and SHG microscopy

CARS microscopy was performed on a custom-built laser scanning microscope employing galvanometric scanning mirrors. Here, the typical pixel dwell time was 31.25 μs with 1024 px × 1024 px per image. Two optical parametric oscillators (OPOs) (Levante Emerald, APE, Berlin, Germany) were pumped by a frequency-doubled 1064 nm Nd:VAN laser (picoTRAIN, High Q laser GmbH, Rankweil, Austria) operating at 80 MHz repetition rate with pulse durations of approximately 7 ps in order to generate the appropriate wavelengths for CARS microscopy. A 60X UPlanSApo water immersion objective (NA = 1.2; Olympus, Hamburg, Germany) was used to focus the spatially and temporally overlapping laser beams into the sample with a focal power of less than 30 mW to avoid the destruction of the biological sample. While collecting the CARS and SHG signals, we placed a stack of filters comprised of a 950 SP, 775 SP, 785 SP and a 514 LP in front of our detector ensuring that only blue-shifted components are being observed (see Fig. S4 in Supplemental Information). In addition, while performing CARS imaging, we expanded this filter set by band-pass filters reducing the spectral window of detection.

In order to image lipids the OPO’s wavelength was tuned to 816.7 nm to serve as the pump beam in combination with the 1064 nm Stokes beam probing the 2845 cm^−1^ CH_2_ stretching vibration. In addition to the filter set, a 660/40 band-pass filter was utilized isolating the generated CARS signal at 660 nm.

For imaging calcium apatite mineral formation, laser wavelengths of 860.6 nm (Stokes) and 796.0 nm (pump) were used to excite the 959 cm^−1^ vibrational mode corresponding to the PO_4_^3−^ stretching vibration generated by the combination of the two OPOs. Here, a tunable band-pass filter at 740/20 nm has been added to the general filter set in order to suppress distracting signals.

SHG was performed by using just the 1064 nm beam with a power of 25 mW in the focal plane and the same general filter set as above in order to suppress the fundamental beam. The nature of the SHG signal was confirmed by independently imaging the collagen distribution in human bone (Fig. 3), mouse skull (Fig. S3), and rat tail (Fig. S2) samples. Due to the infrared nature and the rather long pulse length of the excitation beam, no 2-photon excited autofluorescence was observed in these samples.

All signals were detected using a photomultiplier tube (PMT) (H 9656-20 MOD, Hamamatsu Photonics, Japan) by collecting the photons in forward direction through a 40X LUMPlanFLN objective (NA = 0.8; Olympus, Hamburg, Germany). The resulting PMT signal is measured by an analogue-to-digital (A/D) converter (PCI-6110S, National Instruments, USA) and exploited by ScanImage 3.8, Howard Hughes Janelia Farm Research Campus^30^.

### Processing of CARS and SHG images

All CARS and SHG images were normalized with respect to the intensities of the Pump and Stokes laser beams, the signal integration time, and the amplification factor of the PMT detectors. The images leading to the highest signal contrast (day 21) were then normalized to a maximum value of 1000 and all images normalized relative to this value. This procedure was applied to the lipid, apatite and SHG signals obtained for CARS and SHG experiments.

In a second step, a small pixel shift occurring due to the subsequent acquisition of the different CARS images and SHG images was manually compensated for by pixel-wise shifting the images until the non-resonant components of the lipid and apatite CARS images matched. We used a custom-written Matlab script to determine the best correlation between images to guide this process.

In the final step, the non-resonant background contributions in the CARS images were manually subtracted by subtracting an overall offset, followed by subtraction of the non-resonant CARS contribution from the lipid channel multiplied with a constant factor. This constant factor has been chosen by taking the maximum signal value of a sample region where no resonant CARS signal was observed due to the absence of cells.

The non-resonant background in the lipid CARS channel was split from the resonant contribution by determining the strength of the non-resonant signal in cell-free areas, which was used to apply a threshold value, where values below the threshold are retained for the non-resonant contribution, and values above the threshold are used to display the resonant lipid contribution.

All images were then further processed in Fiji by applying a uniform color lookup table, converting the images to RGB images, and saving the images as PNG files. These images were then read with the Open Source software “Inkscape” to produce the final assembly of images and to add scale bars to the images.

## Additional Information

**How to cite this article**: Hofemeier, A. D. *et al.* Label-free nonlinear optical microscopy detects early markers for osteogenic differentiation of human stem cells. *Sci. Rep.* **6**, 26716; doi: 10.1038/srep26716 (2016).

## Supplementary Material

Supplementary Information
