# Supplementary material for: Label-free nonlinear optical microscopy detects early markers for osteogenic differentiation of human stem cells
Source: Sci Rep. 2016 May 26;6:26716. doi: 10.1038/srep26716 (PMC4880889; doi:10.1038/srep26716)
Supplement: Supplementary Information [file srep26716-s1.pdf]

Supplementary Information, Manuscript "Label-free nonlinear optical microscopy detects early markers for osteogenic differentiation of human stem cells"

Authors: Arne D. Hofemeier, Henning Hachmeister, Christian Pilger, Matthias Schürmann, Johannes FW Greiner, Lena Nolte, Holger Sudhoff, Christian Kaltschmidt, Thomas Huser, Barbara Kaltschmidt

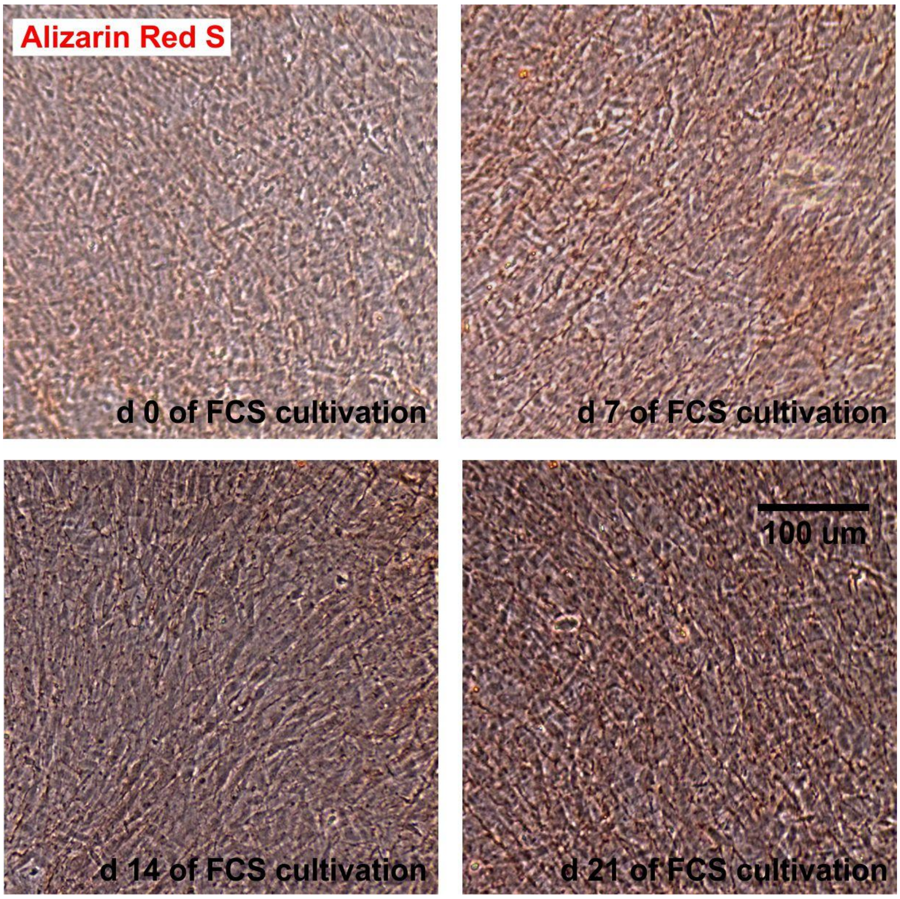

Figure S1. Cultivation of ITSCs under control conditions does not result in the presence of Alizarin Red S-stained calcium deposits. Alizarin Red S staining of ITSCs cultivated for 21 days in medium containing FCS without differentiation supplements revealed no signs of calcium deposition and thus of osteogenic differentiation of ITSCs.

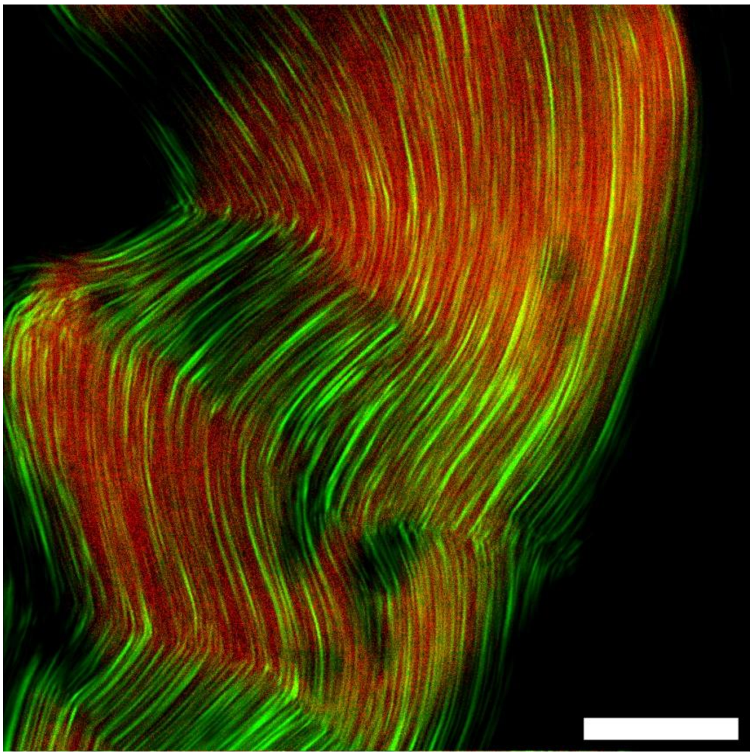

Figure S2. Successful imaging of collagen in mouse tail fiber with SHG microscopy. Next to the unspecific CARS signal at 1662 cm^{-1} (red), the SHG signal of the 1064 nm beam producing a signal at 532nm (green) showed the presence of collagen in mouse tail fiber with its typical fibrillar structure. Scale bar: 30 µm.

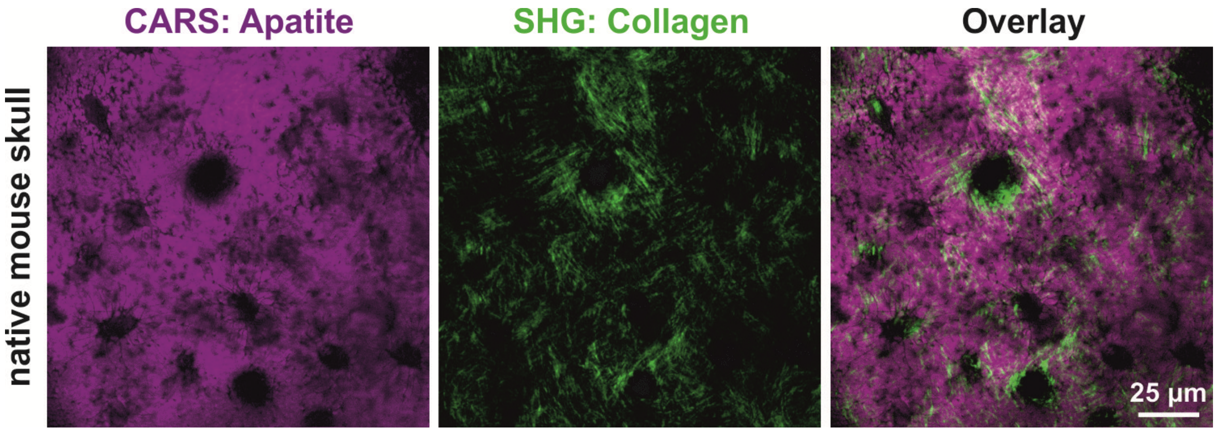

Figure S3. Successful imaging of hydroxyapatite and collagen in mouse skull bone with CARS and SHG microscopy. Mouse skull bone showed a CARS signal (magenta) for calcium hydroxyapatite (959 cm^{-1}) as well as an SHG signal (532 nm) for collagen (green).

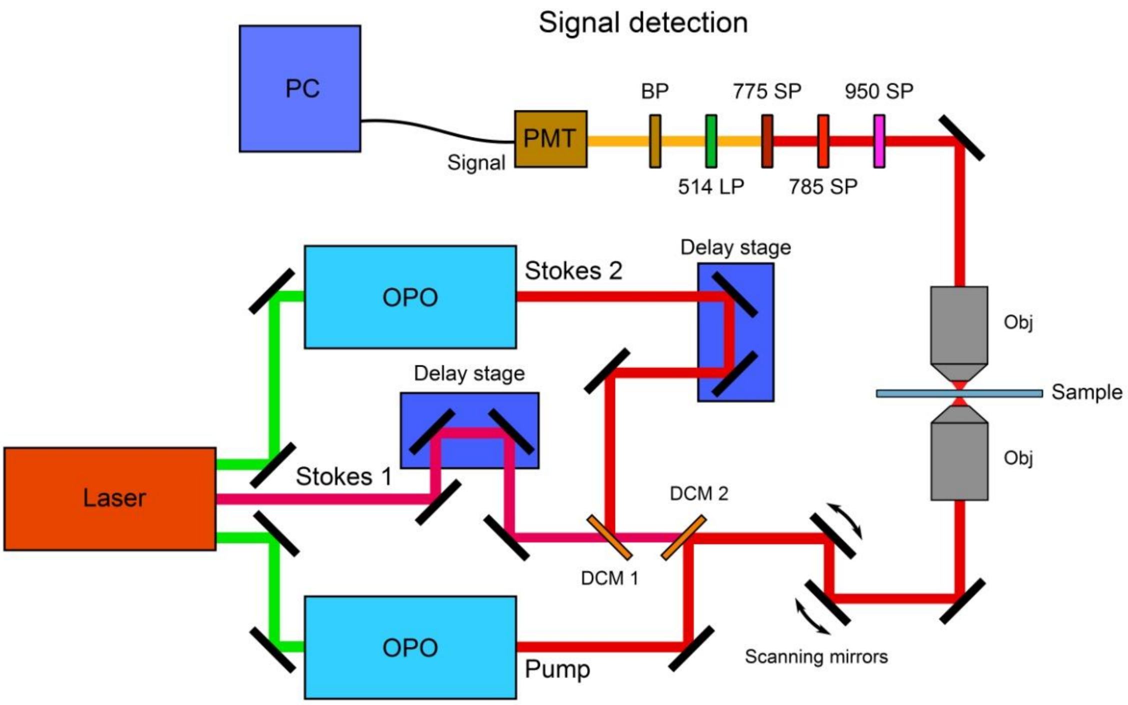

Figure S4. Drawing of the CARS and SHG microscopy setup. The setup consists of a ps ND:VAN laser providing the Stokes beam for the CARS experiment as well as the pump source for the two OPOs. All three beams have been overlapped temporally and spatially using delay stages and dichroic mirrors (DCM1 & DCM2). After the combination, they are directed into the scanning microscope and focused into the sample by an objective lens (Obj). The signal has been collected by a second objective lens and directed to the photomultiplier tube (PMT). In order to suppress the fundamental beams, a filter stack was utilized consisting of a 950 shortpass (SP), a 785 SP, a 775 SP and a 514 longpass (LP) filter to reduce the spectral window of detection. Furthermore, while addressing a specific CARS resonance, an additional bandpass filter was inserted to further narrow the spectral transmission. Finally, the signal output of the PMT was recorded by a PC using an analog-to-digital converter card.
